# Sim-to-Real Domain Adaptation for Early Alzheimer’s Detection from Handwriting Kinematics Using Hybrid Deep Learning

**DOI:** 10.3390/s26010298

**Published:** 2026-01-02

**Authors:** Ikram Bazarbekov, Ali Almisreb, Madina Ipalakova, Madina Bazarbekova, Yevgeniya Daineko

**Affiliations:** 1 Department of Computer Engineering, International IT University, Almaty 050040, Kazakhstan; m.ipalakova@iitu.edu.kz (M.I.); y.daineko@satbayev.university (Y.D.); 2 Department of Engineering, International University of Sarajevo, 71210 Sarajevo, Bosnia and Herzegovina; a.almisreb@iitu.edu.kz; 3 Department of Recreational Geography and Tourism, Al Farabi Kazakh National University, Almaty 050040, Kazakhstan; bazarbekova.madina@kaznu.kz; 4 Department of Electronics, Telecommunications and Space Technologies, Satbayev University, Almaty 050000, Kazakhstan

**Keywords:** Alzheimer’s disease, artificial intelligence, digital biomarkers, handwriting analysis, sensor data, deep learning, health informatics, Sim-to-Real

## Abstract

Alzheimer’s disease (AD) is a progressive neurodegenerative disorder characterized by cognitive and motor decline. Early detection remains challenging, as traditional neuroimaging and neuropsychological assessments often fail to capture subtle, preclinical changes. Recent advances in digital health and artificial intelligence (AI) offer new opportunities to identify non-invasive biomarkers of cognitive impairment. In this study, we propose an AI-driven framework for early AD based on handwriting motion data captured using a sensor-integrated Smart Pen. The system employs an inertial measurement unit (MPU-9250) to record fine-grained kinematic and dynamic signals during handwriting and drawing tasks. Multiple machine learning (ML) algorithms—Logistic Regression, Support Vector Machine (SVM), Random Forest (RF), and k-Nearest Neighbors (kNN)—and deep learning (DL) architectures, including one-dimensional Convolutional Neural Networks (1D-CNN), Long Short-Term Memory (LSTM), and a hybrid CNN-BiLSTM network, were systematically evaluated. To address data scarcity, we implemented a Sim-to-Real Domain Adaptation strategy, augmenting the training set with physics-based synthetic samples. Results show that classical ML models achieved moderate diagnostic performance (AUC: 0.62–0.76), while the proposed hybrid DL model demonstrated superior predictive capability (accuracy: 0.91, AUC: 0.96). These findings underscore the potential of motion-based digital biomarkers for the automated, non-invasive detection of AD. The proposed framework represents a cost-effective and clinically scalable informatics solution for digital cognitive assessment.

## 1. Introduction

Alzheimer’s disease (AD) stands as a defining global health challenge of our time. It is the primary driver of dementia, responsible for roughly 60–70% of cases worldwide and affecting an estimated 55 million individuals, a number projected to triple by 2050 [1]. The disease is characterized by a relentless accumulation of amyloid-beta plaques and tau tangles, triggering a cascade of synaptic failure and neuronal loss [2]. Beyond the biological devastation, AD imposes a staggering emotional and economic toll on caregivers and healthcare systems as patients gradually lose their memory, cognitive faculties, and functional independence. In the absence of a cure, the current clinical imperative is early detection, which provides a critical window for interventions that can delay progression and sustain quality of life [3].

Unfortunately, achieving an early diagnosis remains difficult in clinical practice. Established diagnostic methods, such as magnetic resonance imaging (MRI) [4,5], positron emission tomography (PET) [6] and electrical encephalography (EEG) [7,8,9], provide precise biological data but are often too expensive or invasive for routine screening. Conversely, standard cognitive tests like the MMSE and MoCA are widely accessible but frequently lack the sensitivity to detect subtle impairments in the initial stages of the disease [10]. This limitation highlights a critical need for digital biomarkers which are objective, cost-effective metrics that can bridge the gap between expensive imaging and subjective questionnaires.

Handwriting kinematics represents a particularly promising avenue for such assessment [11]. Writing is a complex cognitive-motor task that requires the integration of fine motor skills, visuospatial perception, and executive planning. Evidence suggests that motor control often begins to degrade before cognitive decline becomes apparent. Patients with early-stage AD frequently exhibit distinct graphomotor patterns, such as micro-tremors, velocity instability (jerkiness), and increased in-air hesitation. With the advent of IoT-based smart pens containing inertial measurement units (IMUs), these subtle kinematic changes can now be quantified with high precision.

The high-dimensional nature of this sensor data presents a compelling case for AI. While traditional Machine Learning (ML) classifiers, such as Support Vector Machines (SVM), have shown promise, they typically rely on handcrafted features, which risks overlooking complex, non-linear patterns inherent in the raw signal [12]. Deep Learning (DL) architectures, particularly Convolutional (CNN) and Recurrent Neural Networks (RNN) such as LSTM, overcome this by learning feature representations directly from raw inputs. However, the deployment of DL in clinical AD detection faces a significant limitation: the gap between model complexity and data availability. Deep neural networks require large-scale datasets to converge effectively [13]. Yet, specialized clinical datasets for tasks like handwriting are often limited in size (n < 200) due to strict privacy regulations and recruitment challenges. Training high-capacity networks on such sparse data inevitably leads to overfitting, where the model memorizes specific patient artifacts rather than learning generalizable disease markers.

In response to this challenge, we propose a strategy based on Sim-to-Real Domain Adaptation. Rather than being constrained by the scarcity of real-world samples, we expand our training data using synthetic handwriting patterns. Our method involves mathematically reproducing known symptoms of AD, such as adding noise to replicate tremors or altering timing to reflect bradykinesia, to create a diverse synthetic dataset. We propose that by exposing the model to this synthetic data alongside real examples, the network can learn more stable and generalized patterns of disease, preventing the overfitting that typically affects small-sample studies.

This paper presents a rigorous comparative study of machine learning and deep learning architectures, specifically investigating the efficacy of Hybrid CNN-BiLSTM model. These architectures are purposefully designed to extract local kinematic anomalies via convolutional layers targeting features like jerk while simultaneously capturing temporal dependencies via recurrent layers.

The primary contributions of this research can be summarized as follows:

1. Synthetic Data Generation Pipeline: We demonstrate a novel methodology for generating realistic synthetic sensor data to effectively mitigate the scarcity of pathological handwriting samples.
2. Validation of Sim-to-Real Transfer: We empirically prove the efficacy of a Domain Adaptation strategy, showing that models trained on a hybrid mix of synthetic and real data significantly outperform those trained on real clinical data alone.
3. Architectural Benchmarking: We provide a comprehensive evaluation of deep learning models on 18-channel engineered kinematic features, establishing a new baseline for accuracy (>90%) and specificity (>95%) in this domain.

This paper is organized as follows: Section 2 provides review for existing research in the field. Section 3 introduces our methodology, detailing the mathematical basis of the Sim-to-Real pipeline and the architecture of the Hybrid CNN-BiLSTM. In Section 4, we validate the model through experimental comparisons against baseline classifiers. Finally, Section 5 discusses the clinical implications and limitations of the study, followed by concluding remarks in Section 6.

## 2. Related Work

The field of automated diagnosis for neurodegenerative diseases has shifted significantly over the last decade. Research has moved from simple statistical analysis of drawing tablets to complex deep learning models using wearable sensors. This section reviews this progression, analyzing the strengths and specific failures of existing methods that led to the development of our Sim-to-Real approach.

Formally, we define the diagnostic task as follows: Given a dataset $D=\left\{{\left(X_{i},y_{i}\right)}_{i=1}^{N}\right\}$, where the input $X_{i}\in R^{T\times C}$ represents the multivariate time-series of pen motion (C=18 channels) and the output $y_{i}\;\in \left\{0,1\right\}$ denotes the clinical diagnosis, our objective is to approximate a mapping function $f:\;R^{T\times C}\;\to \left[0,\;1\right]$ that minimizes the generalization error on unseen real-world patients by leveraging synthetic domain knowledge.

### 2.1. Handcrafted Features and Classical Machine Learning

For years, the clinical standard for digital handwriting analysis relied on digitizer tablets (e.g., Wacom) combined with classical ML. The workflow was predominantly manual: a patient performs a task, and researchers engineer global statistical descriptors. Werner et al. established a baseline, utilizing ANOVA to demonstrate that kinematic variables, specifically mean writing pressure and velocity, decrease significantly as cognitive decline progresses from Mild Cognitive Impairment (MCI) to AD [14]. Rosenblum et al. expanded this by analyzing in-air movements, revealing that prolonged non-writing pauses are often a more sensitive biomarker of executive dysfunction than the writing itself [15]. To automate diagnosis, researchers applied supervised classifiers to these features. Drotár et al. (2016) achieved an accuracy about 80% using SVM on spiral drawing tasks, isolating jerk as a key indicator of motor control loss [16]. Similarly, Pereira et al. employed Random Forests to quantify tremor intensity. Although interpretable, these methods depend heavily on manual feature extraction [17]. By reducing complex time-series data to single scalar values (e.g., mean velocity), they lose the detailed temporal dynamics where transient pathological events, such as momentary micro-tremors or hesitations occur.

### 2.2. Wearable Sensors and Time-Series Analysis

Digitizer tablets are limited by their stationary nature and restriction to 2D surface coordinates. Real-world pathological tremors, however, occur in 3D space. This limitation drove the adoption of wearable inertial measurement units (IMUs) and instrumented Smart Pens. Kourtis et al. highlighted the opportunity of using mobile and wearable devices for digital biomarkers [18]. Unlike tablet studies, wearable sensors analyze the raw acceleration signal directly. Findings have confirmed that spectral analysis of the inertial signal can detect high-frequency micro-tremors (4–12 Hz) invisible to the naked eye. However, the high dimensionality of sensor data, often sampled at >100 Hz across 6 axes, complicates analysis for classical models. In our previous studies, we reviewed artificial intelligence methods for AD diagnosis, noting the insights gained from sensor data analysis but also the challenges in processing such complex signals [19]. Park et al. applied LSTM networks for similar sensor-based gait analysis in Parkinson’s disease [20]. Although LSTMs can model temporal dependencies, the authors noted that the models struggled with signal noise and required extensive pre-processing to converge, underscoring the challenges of applying raw deep learning to noisy biological signals.

### 2.3. Deep Learning Architectures

To address the limitations of manual feature extraction and noise sensitivity, the field has increasingly adopted DL.

*Convolutional Neural Networks (CNNs)*: Originally designed for image recognition, CNNs have been adapted for 1D time-series classification. Babu et al. proposed a novel approach by transforming dynamic signature velocity profiles into 2D image representations and processing them with a CNN [21]. They achieved high verification rates, but their dataset was limited to fewer than 60 subjects, raising concerns about overfitting. Taking a different approach, Cilia et al. (2022) established a comprehensive benchmark for on-line handwriting, evaluating various CNN architectures on a novel dataset (DARWIN) [11]. While their deep learning models achieved competitive results, they highlighted that standard CNNs often struggle to fully capture long-term temporal dependencies compared to recurrent architectures. This limitation suggests that while CNNs are powerful feature extractors, they require integration with sequence models for optimal performance. Kang et al. (2024) proposed a novel approach for early Alzheimer’s diagnosis via handwriting using self-attention mechanisms to capture long-range dependencies [22]. They achieved high verification rates, addressing the risk of overfitting common in small datasets.

*Hybrid Architectures*: Hybrid models have gained popularity because they combine two strengths: detecting short-term anomalies such as tremors and understanding long-term patterns such as fatigue. For example, Ordóñez and Roggen (2016) combined CNNs and LSTMs to analyze data from wearable sensors [23]. They showed that the CNN layers effectively clean the signal, acting as a filter before the LSTM processes the timeline. Similarly, El-Maachi et al. (2020) used this approach to assess Parkinson’s severity from video data, achieving much better results than standard models [24]. Qi et al. conducted a study of assisted screening for Alzheimer’s disease based on handwriting and gait analysis, demonstrating the efficacy of combining multiple modalities [25]. However, this powerful combination has rarely been tested on handwriting data for Alzheimer’s detection. Our study aims to apply this method to see if it improves the analysis of fine motor skills.

### 2.4. The Data Scarcity and Sim-to-Real Adaptation

Despite recent architectural advancements, the application of Deep Learning in clinical settings is often limited by the lack of large datasets. Bazarbekov et al. emphasized in their review that deep networks typically require large-scale data to learn generalized representations, a requirement rarely met in clinical AD studies [26]. Clinical handwriting datasets are usually small. Training high-capacity models on such sparse data leads to memorization rather than learning. To mitigate this, Data Augmentation (DA) is essential. While techniques like Jittering and Scaling are effective regularizers, more advanced generative approaches are needed. Most recently, Ahmed et al. proposed using Variational Autoencoders to augment handwriting datasets, achieving high accuracy [27]. However, generative models often operate as black boxes. Our study introduces a deterministic, robust alternative: Sim-to-Real Domain Adaptation. Instead of unstable generative models, we use mathematical modeling to generate a massive dataset of synthetic handwriting kinematics that mimics specific pathological features such as stochastic tremor injection and non-linear time warping. This approach allows us to pre-train a complex Hybrid CNN-BiLSTM on larger data using synthetic samples and fine-tune it on the limited real-world data, effectively solving the small sample size problem. A comparison of these diagnostic frameworks across different modalities like MRI, EEG and Kinematics, highlighting the specific limitations addressed by our proposed approach is presented in Table 1.

## 3. Materials and Methods

**Figure 1:**
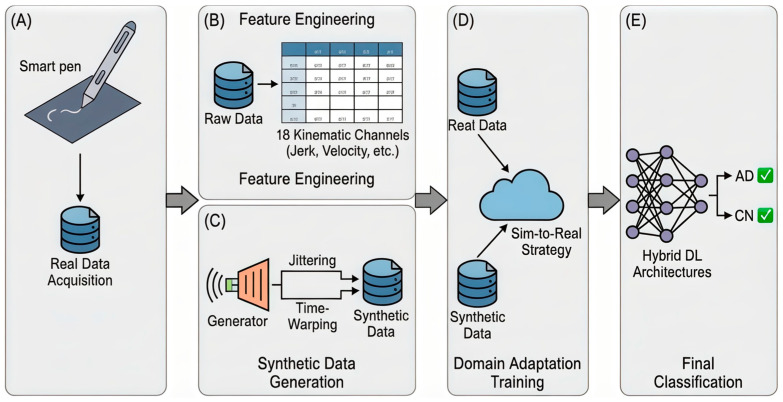
Operational workflow of the proposed Sim-to-Real framework. The workflow starts with Data Acquisition (**A**), where a smart pen records handwriting movements. These raw signals move to Feature Engineering (**B**), where they are converted into 18 kinematic channels to highlight specific motor patterns. To help with the limited amount of real data, the Synthetic Data Generation (**C**) module uses a physics model to create simulated data by adding tremors and hesitation effects. In the Domain Adaptation Training (**D**) stage, this simulated data is mixed with real patient data to improve training. Finally, the Hybrid Deep Learning Architecture (**E**) uses this combined information to distinguish between Alzheimer’s Disease and healthy conditions.

In this study, we propose a novel framework for the early detection of Alzheimer’s disease (AD) that addresses a critical challenge in medical informatics: the lack of large, labeled clinical datasets. Our methodology is built upon the Sim-to-Real Domain Adaptation concept. We integrate advanced synthetic data generation techniques with a hybrid deep learning architecture to analyze handwriting kinematics captured via a smart pen. The complete workflow of our approach is illustrated in Figure 1, comprising data acquisition, feature engineering, synthetic sample generation, and domain adaptation training.

### 3.1. Data Collection and Acquisition

Participants were recruited through institutional collaborations coordinated by United Brain Centre LLP (Kazakhstan), under the leadership of its director, who is one of the few professionals in advancing AD research in the region. The study cohort consisted of 106 individuals clinically diagnosed with AD and 109 Cognitively Normal (CN) group.

AD diagnoses were established following internationally recognized criteria and confirmed via neuroimaging (MRI and/or PET) and cognitive evaluation using the Montreal Cognitive Assessment (MoCA) [28]. All AD participants were on mild cognitive decline (MCI) stage and had a mean age of 65 years with range from 58 to 82 years. CN participants were matched to the AD group by age and education level, with MoCA scores used to confirm the absence of cognitive impairment.

Recruitment of CN participants was performed through community outreach to minimize selection bias. All participants provided written informed consent, and the study protocol was approved by the Institutional Ethics Committee, in accordance with the Declaration of Helsinki [29].

**Figure 2:**
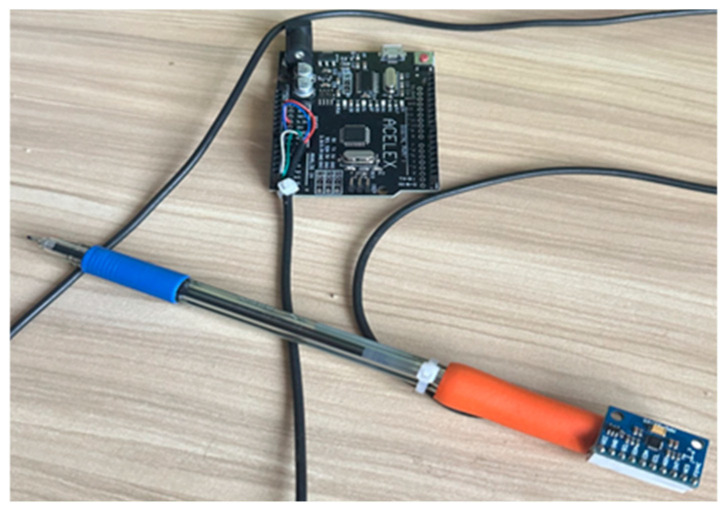
Prototype of Smart handwriting tool.

Handwriting motion data were collected using a custom-designed Smart Handwriting Tool (Smart Pen) (Figure 2), engineered to replicate the weight, geometry, and ergonomic comfort of a conventional pen. The device equipped with an embedded 9-axis Inertial Measurement Unit (IMU) MPU-9250 (InvenSense, San Jose, CA, USA). Unlike traditional digitizer tablets that only record 2D positional data (x, y), this device captures the full 6-degrees-of-freedom motion dynamics of the hand. The sensor configuration was defined as follows:

1. Angular velocity *g*(*t*): Represents the rate of rotation around the principal axes, measured in degrees per second (°/s):

(1) $$g(t)=[g_{x}(t),g_{y}(t),g_{z}(t){]}^{⊤}(\pm 2000{}^{\circ}/s),$$

2. Linear acceleration *A*(*t*): Represents the specific force acting on the pen in three-dimensional space, measured in units of $g\;\left(9.8\frac{m}{s^{2}}\right):$

(2) $$A(t)=[a_{x}(t),a_{y}(t),a_{z}(t){]}^{⊤}(\pm 2g\;to\;\pm 16g),$$

3. Magnetic field *m*(*t*): Represents the absolute orientation relative to the Earth’s magnetic north

(3) $$m(t)=[m_{x}(t),m_{y}(t),m_{z}(t){]}^{⊤},$$

A Digital Motion Processor (DMP) was used for on-device sensor fusion, improving spatial orientation and reducing drift errors. Signals were sampled at 100 Hz, with a ±2000°/s range for the gyroscope and ±16 g for the accelerometer. A 25 Hz low-pass filter was applied to suppress high-frequency noise. Data were transmitted via USB using CoolTerm software (v2.4.0) [30], ensuring lossless and synchronized acquisition.

Participants were seated comfortably at a standard desk in a quiet environment. They were instructed to perform a standardized graphomotor protocol using the instrumented Smart Pen on standard A4 paper. The protocol was designed to elicit different cognitive-motor loads:

1. Overlearned Task (Sentence Writing): Participants wrote a standard, familiar sentence which covers all letters in Kazakh alphabet. This task assesses automated motor programs, where deterioration often manifests as reduced velocity and increased variability.
2. Visuospatial Task (Archimedean Spiral Drawing): Participants drew a spiral from the center outward. This task heavily engages visuospatial integration and fine motor planning, often revealing tremors and trajectory irregularities.
3. Repetitive Task (Oscillating Loops): Participants drew continuous cursive loops. This assesses rhythmic motor control and susceptibility to fatigue (bradykinesia).

No time constraints were imposed, allowing participants to write at their natural pace.

### 3.2. Data Preprocessing

Raw signals collected from inertial sensors are rarely perfect, they often contain electronic noise and hardware artifacts that can confuse deep learning models. To transform this raw input into a clean dataset ready for analysis, we applied a standard four-step preprocessing pipeline:

1. Noise Filtering: First, we applied a 25 Hz low-pass filter to all sensor channels. This specific frequency was chosen to strip away high-frequency electronic jitter and involuntary muscle artifacts, while keeping the genuine, meaningful dynamics of handwriting intact.
2. Gravity Compensation: An accelerometer does not just measure hand movement; it also constantly measures the Earth’s gravity. To isolate the user’s actual motor intent, we subtracted the static gravity component from the raw acceleration data. This ensures the model analyzes the hand’s motion, not the pull of the Earth.
3. Normalization: Every participant writes differently, some use large, sweeping strokes, while others write with small, contained movements. To ensure our neural network focuses on the patterns of movement rather than the amplitude of the signal, we scaled all 18 kinematic channels to a fixed (0, 1) range using Min-Max Normalization:
  (4) $$x_{norm}=\frac{x-\min\left(x\right)}{\max\left(x\right)-\min\left(x\right)}$$
4. Sequence Padding: Finally, since some participants write faster than others, the duration of the tasks varied. Deep learning models require inputs of uniform size, so we standardized the sequence length. All recording samples were padded or truncated to a fixed length of T = 1084 time steps. This length was sufficient to cover the full duration of the tasks without losing critical data.

### 3.3. Feature Engineering

Raw sensor data alone streams of numbers representing acceleration and rotation are often too noisy and abstract for a neural network to interpret effectively [31]. To bridge the gap between raw signals and clinical insights, we performed Feature Engineering (Block B in Figure 1). This process transforms the 9-axis IMU data into meaningful biomarkers that reflect the specific motor degradations associated with Alzheimer’s Disease (AD), such as bradykinesia (slowness), tremors, and loss of fine motor control.

The data collected from the MPU-9250 sensor provided us with three core components: linear acceleration $A\left(t\right)=\left[A_{x},\;A_{y}{,\;A}_{z}\right]$ angular velocity $\omega \left(t\right)=\left[\omega_{x},\omega_{y},\omega_{z}\right]$, and magnetic field orientation $m\left(t\right)=\left[m_{x},m_{y},m_{z}\right].$ However, we excluded the magnetometer data from our feature set due to its high susceptibility to electromagnetic interference typical of indoor clinical environments, which renders absolute heading unreliable for fine motor analysis. As a result, we used only the 6-DOF inertial sensors. To capture the movement dynamics, we expanded these inputs into 18 Kinematic Channels (shown in Table 2) designed to detect motor decline:

1. Velocity and Trajectory Reconstruction: Since standard accelerometers are prone to drift errors over time, we used the gyroscope data to stabilize the signals. By integrating the acceleration over time, we reconstructed the velocity profiles of the pen tip.
  (5) $$v_{t}=v_{t-1}+a_{t}\cdot ∆t$$

This is crucial because AD patients often exhibit reduced peak velocity and inconsistent writing speeds compared to healthy controls.

2. Jerk Analysis (Smoothness): We calculated the rate of change in acceleration to measure movement stability below. This metric is critical for detecting neurodegeneration, as it quantifies the micro-tremors and abrupt motor corrections that distinguish the unstable handwriting of AD patients from the fluid strokes of healthy controls.
  (6) $$J\left(t\right)=\frac{dA\left(t\right)}{dt}$$

3. Rotational Dynamics: We derived Rotational Acceleration to quantify rapid changes in angular velocity. This feature captures wrist instability and the specific rotational tremors that manifest during curvilinear motor tasks.
  (7) $$\alpha =\frac{\omega_{t}-\omega_{t-1}}{∆t}$$

4. Spatial Orientation: We obtained the pen’s 3D posture using the sensor’s DMP. By handling sensor fusion internally via on-chip processing, the DMP filters out gyroscopic drift in real-time, providing robust Euler angles (Roll(θ), Pitch(φ), Yaw(ψ)) without the need for complex post-processing of raw data.

### 3.4. Sim-to-Real: Synthetic Data Generation and Domain Adaptation

Deep learning models typically require large datasets to generalize effectively [32,33,34,35,36]. Our clinical dataset contains 215 participants, which is relatively small for training complex neural networks and increases the risk of overfitting. To address this, we implemented a Sim-to-Real Domain Adaptation strategy (Block C in Figure 1).

We expanded our training dataset by generating a total of 300 synthetic samples (150 for the AD group and 150 for the CN group). While extending the dataset with real healthy subjects is theoretically possible, applying synthetic augmentation to both classes was methodologically critical to prevent source bias.

If we had generated synthetic data exclusively for the AD group, the deep learning model might have incorrectly learned to associate the specific artifacts of synthetic generation with the disease label. By applying Sim-to-Real augmentation to both Healthy and Alzheimer’s classes, we ensured that the model focuses on clinically relevant kinematic features such as tremor and hesitation rather than discriminating between real and synthetic data sources.

To replicate the high-frequency kinetic tremors and loss of fine motor control characteristic of AD, we modeled tremor as stochastic noise injected into the acceleration and jerk channels. For a given kinematic time-series channel $x\left(t\right),$ the augmented signal $x_{tremor}\left(t\right)$ is defined as:

(8) $$x_{tremor}\left(t\right)=x\left(t\right)+\xi \left(t\right),\;\xi \left(t\right)∼N\left(0,\sigma^{2}\right)$$

where $\xi \left(t\right)$ represents additive white Gaussian noise drawn from a normal distribution N with zero mean. The standard deviation *σ* serves as a hyperparameter controlling tremor intensity. We varied σ uniformly within the range $\left[0.01,\;0.05\right]$ relative to the signal amplitude, simulating a spectrum of severity from mild physiological tremor to pronounced pathological shaking.

Cognitive decline often manifests as bradykinesia and irregular processing delays during motor planning. To simulate this without altering the spatial geometry of the handwriting, we applied non-linear Time-Warping. Let t be the original time index and $\tau \left(t\right)$ be a monotonic warping function. The warped signal $x_{slow}\left(t\right)$ is obtained by resampling the original signal at new temporal locations:

(9) $$x_{slow}\left(t\right)=x\left(\tau \left(t\right)\right)$$

**Figure 3:**
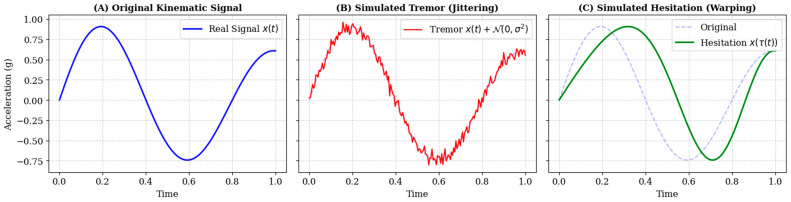
Examples of signal augmentation. (**A**) Original time-series. (**B**) Result of noise injection to simulate tremors. (**C**) Result of time-warping to simulate irregular writing rhythm.

The warping function $\tau \left(t\right)$ is generated via cubic spline interpolation through K randomly perturbed knot points along the temporal axis. This process locally stretches specific stroke segments (simulating hesitation) while compressing others, strictly preserving the sequence of motor events while altering their rhythm (Figure 3). Crucially, to prevent the model from distinguishing classes based solely on the presence of synthetic artifacts, probabilistic Time-Warping was applied to both the AD and CN groups. While AD samples were warped to specifically simulate bradykinesia, CN samples were subjected to random mild warping. This ensures that the deep learning model learns distinct pathological temporal features rather than simply detecting the augmentation process itself.

The synthetic dataset $D_{syn}$ effectively expands the training distribution, exposing the network to a wide range of motor anomalies that are underrepresented in the small clinical cohort.

### 3.5. Classification Models and Training Protocol

To validate the effectiveness of our proposed framework, we conducted a rigorous comparison between established machine learning algorithms and our Hybrid Deep Learning architecture.

#### 3.5.1. Baseline Machine Learning Classifiers

To establish a solid performance benchmark, we evaluated four traditional supervised learning algorithms: LR, SVM, RF, and k-NN.

We recognized that simple default settings often lead to suboptimal performance. Therefore, to ensure a fair competition against our deep learning model, we performed a systematic hyperparameter optimization shown in Table 3 for each baseline using Grid Search with 3-fold stratified cross-validation. Before training, all features were standardized using Z-score normalization to ensure that high-magnitude signals such as angular velocity did not dominate the objective function.

The specific configurations were chosen to balance model complexity with generalization:

1. Logistic Regression: We tested various regularization strengths $\left(C\;\in \;\{0.01,\;0.1,\;1,\;10\}\right)\;$ using the liblinear solver, which is well-suited for smaller datasets.
2. SVM: We utilized a Radial Basis Function (RBF) kernel to capture non-linear relationships in the kinematic data. We tuned both the regularization parameter (C∈ {0.1, 1, 10}) and the kernel coefficient $\left(\gamma \;\in \;\{’scale’,auto’\right\})$.
3. Random Forest: As an ensemble method, we explored different numbers of estimators $\left(50,\;100\right)$ and constrained the maximum tree depth $\left(5,\;10\right)$ to prevent the model from memorizing noise in the training data (overfitting).
4. k-NN: We experimented with neighborhood sizes $k\;\in \;\left\{3,\;5,\;7,\;9\right\}$ to find the optimal balance between local sensitivity and noise smoothing.

#### 3.5.2. Hybrid Deep Learning Architecture

To address the limitations of classical models, we designed a Hybrid Neural Network (Block E in Figure 1) capable of analyzing handwriting as a dynamic sequence rather than a static image. The architecture described in Table 4, consists of two sequential blocks:

1. Local Feature Extraction (1D-CNN): The input sequence first flows through convolutional layers. These layers act as local filters, scanning the signal to detect short-term anomalies, such as sudden spikes in jerk or momentary tremors, which are often invisible in global statistics.
2. Global Context Analysis (BiLSTM): The features extracted by the CNN are then processed by Bidirectional LSTM units. These units analyze the writing rhythm in both forward and backward directions, allowing the model to distinguish between a natural pause and a pathological hesitation characteristic of Alzheimer’s disease.

*Training Protocol and Domain Mixing*: We observed that models trained exclusively on synthetic data yielded lower accuracy when tested on real clinical subjects. This confirms that synthetic samples, while capturing general motor patterns, do not fully reproduce the specific variability of real-world handwriting.

To address this limitation, we adopted a Mixed Supervision Strategy:

1. *Training Set*: We augmented the synthetic dataset (300 participants) with a subset of 80% of the real clinical data (~172 participants). These real samples serve as reference points, helping the model align the synthetic features with the actual distribution of patient data.
2. *Testing Set*: The remaining 20% of the real clinical data (~43 participants, balanced between AD and CN groups) was strictly held out for testing. This ensures that the reported performance reflects the model’s ability to generalize to new patients, rather than memorizing the training set.

We evaluated this approach using 5-Fold Cross-Validation to ensure the results were stable and reproducible.

## 4. Results

In this section, we present the evaluation of the proposed Sim-to-Real framework. We analyze the model’s diagnostic performance using stratified cross-validation, examine the types of errors via confusion matrices, and conduct a direct comparison with traditional machine learning baselines to demonstrate the advantages of the hybrid architecture.

### 4.1. Performance of the Hybrid Sim-to-Real Model

The primary objective was to distinguish between patients with early-stage Alzheimer’s Disease (AD) and Cognitively Normal (CN) using the generated Sim-to-Real model.

*Stability and Accuracy*:

The experimental results from the 5-fold cross-validation are summarized in Table 5. The proposed Hybrid CNN-BiLSTM model achieved consistent performance across all folds, with an average AUC of 0.963 (±0.01) and an overall classification Accuracy of 91.2%.

**Figure 4:**
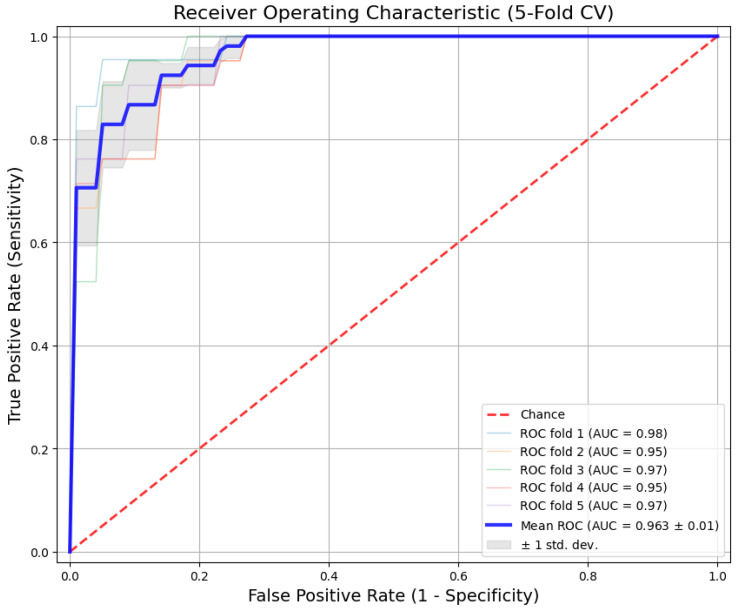
Mean Receiver Operating Characteristic (ROC) curves for the proposed Hybrid CNN-BiLSTM model. The solid blue line represents the average performance across all 5 folds for this specific architecture, with the shaded area indicating the standard deviation.

Figure 4 shows the ROC curves for each fold. To avoid selection bias, we report the mean performance where the curves are tightly clustered around the mean, with very low variance. This indicates that the model is robust and its diagnostic power remains stable regardless of how we split the data into training and testing sets.

### 4.2. Confusion Matrix and Clinical Safety

**Figure 5:**
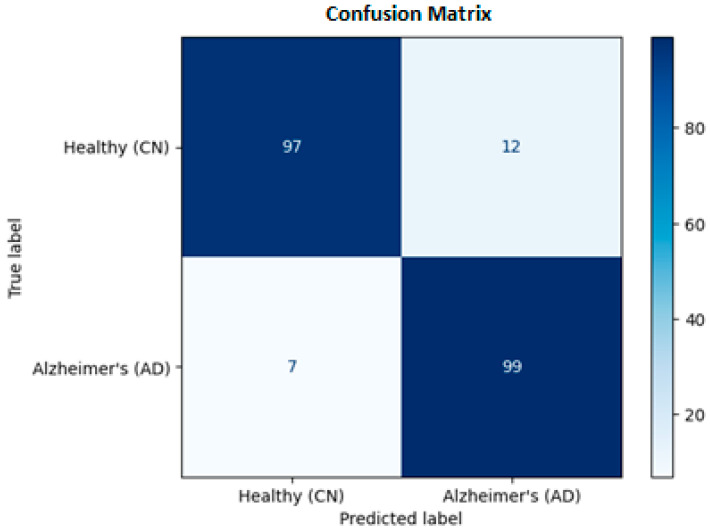
Aggregated Confusion Matrix for the proposed Hybrid CNN-BiLSTM model.

To evaluate the clinical utility of the model, we analyzed the aggregated Confusion Matrix (Figure 5), which combines predictions from all five test folds.

1. True Positives: The model correctly identified 99 AD patients.
2. False Negatives: It missed only 7 cases.
3. True Negatives: It correctly confirmed 97 healthy subjects.
4. False Positives: It incorrectly flagged 12 healthy subjects.

From a medical perspective, Sensitivity (Recall) is the most critical metric, as missing a positive diagnosis is dangerous. Our model achieved a Sensitivity of 93.4%, meaning it successfully detected the majority of patients. The slight tendency to over-predict disease (12 False Positives) is acceptable for a screening tool, where safety is the priority.

### 4.3. Comparison with Baseline Machine Learning Models

To validate the need for a complex Deep Learning architecture, we compared our results against the optimized baseline models (SVM, Random Forest, Logistic Regression, KNN) described in Section 3.5.1. These models were trained on the same real-world dataset using statistical features.

**Figure 6:**
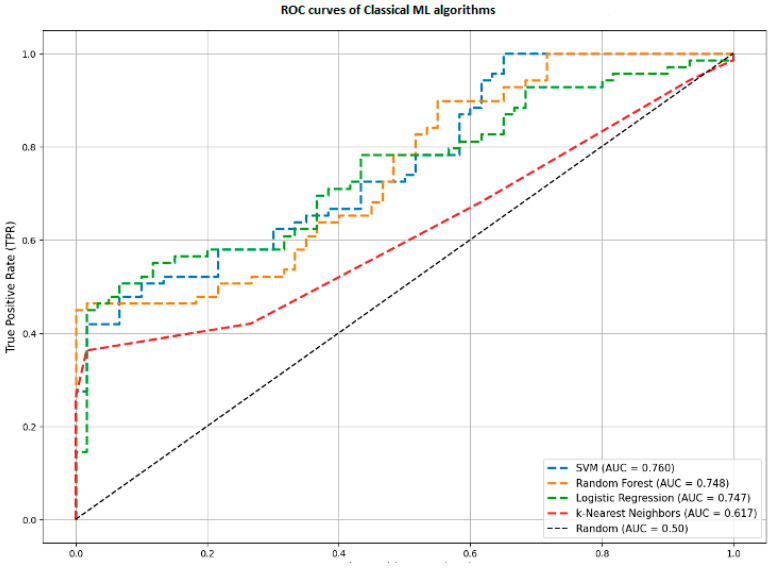
Comparative analysis of ROC curves for Classical ML algorithms.

Figure 6 reveals a significant performance gap:

1. Proposed Hybrid Model: AUC = 0.966.
2. Support Vector Machine (SVM): AUC = 0.760.
3. Random Forest (RF): AUC = 0.748.
4. K-Nearest Neighbors (KNN): AUC = 0.617.

This comparison highlights a fundamental limitation of classical machine learning for this task. Models like Random Forest or SVM rely on aggregated statistics such as average velocity or maximum jerk. They view handwriting as a static set of numbers.

In contrast, our Hybrid CNN-BiLSTM architecture analyzes handwriting as a dynamic sequence. It can detect specific temporal events such as a momentary tremor during a curve or a hesitation before a stroke. This ability to capture the historical data of the movement allows the hybrid model to detect subtle signs of AD that are lost when data is averaged into simple statistics.

## 5. Discussion

This study aimed to develop a non-invasive, scalable tool for the early detection of AD using handwriting kinematics. The main challenge in this field is the lack of large clinical datasets, which hinders the training of reliable Deep Learning models. By implementing a Sim-to-Real framework, we successfully trained a Hybrid CNN-BiLSTM model that achieved an AUC of 0.963, significantly outperforming traditional machine learning baselines.

The performance disparity between the hybrid model and classical baselines (AUC 0.963 vs. 0.760) indicates that the motor symptoms of AD are inherently temporal. Classical models (SVM, RF) failed to exceed an AUC of 0.760 because they utilize aggregated statistics, effectively averaging out transient pathological events. In contrast, the BiLSTM layers in our architecture identified specific, localized anomalies, such as micro-tremors or hesitations, that occur only during complex motor execution.

The generation of 300 synthetic samples prevented overfitting, a common failure mode when training deep networks on small cohorts. These synthetic samples established a baseline representation of motor degradation. The inclusion of 80% real data during training aligned this representation with the sensor-specific noise characteristics of the physical device, bridging the domain shift between simulated and real-world kinematics.

Current state-of-the-art methods typically rely on standard classifiers and dynamic features, achieving accuracies in the range of 85% [27]. Our method improved this benchmark to 91.2%. Unlike approaches utilizing GANs, which can be unstable for multivariate time-series data, our use of deterministic mathematical modeling such as Time-Warping and Noise Injection, ensures reproducibility. This approach allows for precise control over the simulated severity of motor impairment, providing a transparent augmentation process rather than a black box generation.

High Sensitivity (93.4%) is critical for a primary screening tool to minimize false negatives. Our model missed only 7 out of 106 AD cases. While the model produced 12 false positives, this trade-off is clinically acceptable; false positives result in follow-up examinations, whereas false negatives delay necessary intervention. The system demonstrates sufficient reliability for deployment as a decision-support tool in non-specialized clinical settings.

### Clinical Interpretability and Feature Analysis

**Figure 7:**
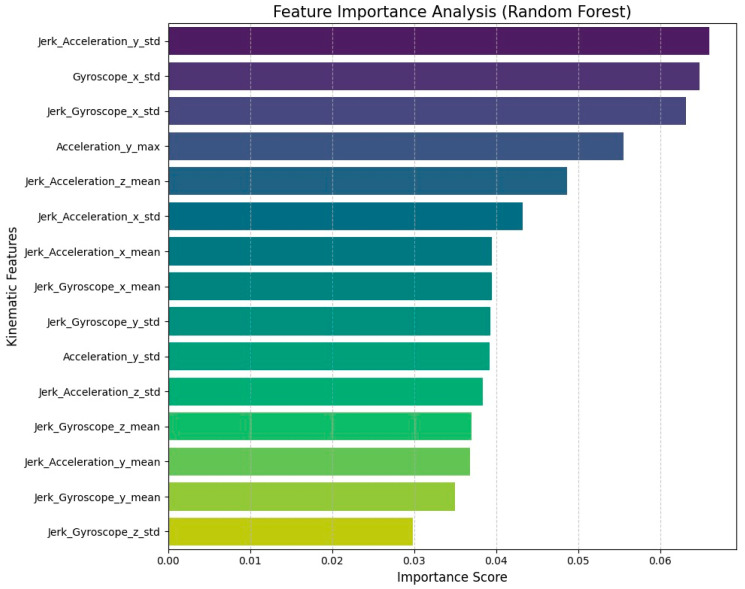
Feature Importance ranking derived from the Random Forest baseline.

To better understand the clinical logic behind the model’s decisions, we examined the feature importance rankings from the Random Forest baseline (Figure 7). The results clearly show that the standard deviation of Jerk and Gyroscope signals are the most powerful predictors. This makes perfect clinical sense, as it mirrors the actual motor symptoms of Alzheimer’s Disease. High variability in jerk captures the micro-tremors and loss of smoothness that distinguish patients from healthy individuals. Furthermore, the model’s heavy reliance on variance metrics suggests it is successfully detecting the irregular movement and hesitation (bradykinesia) typical of cognitive decline. This confirms that our AI is not simply finding patterns in noise, but is prioritizing the same physiological biomarkers used by clinicians, validating the realism of our Sim-to-Real augmentation.

The study has three main limitations. First, the dataset is restricted to a single demographic cohort, necessitating validation on diverse populations to ensure generalizability. Second, the current IMU setup lacks axial pressure sensors. Incorporating pressure dynamics could improve the differentiation between healthy and pathological samples. We anticipate that adding pressure data will help the model distinguish between moments when the hand stops on the paper and moments when the pen is lifted while the patient thinks about the next movement. Since cognitive decline often causes people to hesitate without leaving a trace on the page, measuring these subtle changes in pressure would allow the system to detect these invisible pauses. This capability would significantly refine the detection of early-stage impairments by capturing the full picture of motor planning delays. Third, the current model currently distinguishes only between Alzheimer’s patients and healthy controls. A key direction for future work is to differentiate Alzheimer’s from other neurodegenerative conditions, particularly Parkinson’s Disease, which presents overlapping motor symptoms. We plan to address this by extending our Sim-to-Real framework to simulate the specific motor features of Parkinson’s. By adjusting the physics-based generator to model the characteristic resting tremor, which typically occurs at 4 to 6 Hertz, we can train the model to recognize distinct pathological patterns. This will demonstrate the generalizability of our framework and allow for precise differential diagnosis between different neurodegenerative disorders.

## 6. Conclusions

This study establishes the efficacy of using handwriting kinematics combined with Sim-to-Real Domain Adaptation as a robust biomarker for the early detection of Alzheimer’s Disease. While previous research has largely been constrained by the scarcity of annotated clinical data, our findings demonstrate that physics-based mathematical modeling can effectively generate the prior knowledge required to train complex Deep Learning architectures.

The proposed Hybrid CNN-BiLSTM model, trained on a tailored mix of synthetic and real data, achieved a diagnostic accuracy of 91.2% and an AUC of 0.963 on a held-out test set. These metrics not only surpass traditional machine learning benchmarks but also validate the hypothesis that fine motor control degrades in non-linear, temporal patterns that are best captured by sequence-based networks rather than static statistical analysis.

From a clinical perspective, the system’s high Sensitivity (93.4%) positions it as an ideal candidate for widespread screening in primary care. Unlike expensive neuroimaging (PET/MRI) or invasive lumbar punctures, our approach requires only a low-cost inertial sensor and a standard writing task. This accessibility could significantly reduce the burden on specialized neurological centers by filtering and identifying high-risk individuals at a much earlier stage.

Methodologically, this work contributes a transferable framework for medical AI. We showed that deterministic augmentation, specifically Time-Warping and Noise Injection, provides a transparent and stable alternative to black-box generative models like GANs. By successfully bridging the domain gap between simulated physics and real-world sensor noise, we offer a scalable template that can be adapted for other motor–cognitive disorders, such as Parkinson’s or Huntington’s disease.

Future developments will focus on validating this framework in longitudinal studies to monitor disease progression over time and integrating multi-modal sensors to further refine diagnostic specificity. Ultimately, this research represents a step towards objective, quantified, and accessible digital neurology.

## Figures and Tables

**Table 1:** A comparison of related works in neurodegenerative motion analysis.

Ref.	Study & Year	Data Modality	Methodology	Key Limitation Identified
[4]	Khan et al. (2025)	Three-dimensional MRI	Enhanced ROI-guided Deep Learning	Requires expensive, non-portable equipment; not suitable for mass screening.
[6]	Bente et al. (2025)	Three-dimensional MRI	3D-CNN & Transfer Learning	High computational cost; invasive procedure compared to handwriting.
[13]	Assaduzzaman et al. (2024)	MRI Data	Customized CNN (ALSA-3)	CNNs excel at spatial features but miss the temporal evolution of neurodegeneration.
[7]	Li et al. (2025)	EEG Signals	Functional Connectivity Analysis	Signal acquisition is complex and susceptible to environmental noise artifacts.
[9]	Sugden & Diamandis (2023)	EEG Signals	Deep Learning Classification	Limited interpretability of deep features compared to kinematic biomarkers.
[8]	Santos Toural et al. (2021)	EEG Biomarkers	Statistical Classification	Relies on handcrafted spectral features; less robust to inter-subject variability.
[12]	Sourkatti et al. (2025)	Clinical Records	Feature Selection on Tabular Data	Lacks direct measurement of motor–cognitive decline; depends on historical data quality.
[14]	Werner et al. (2006)	Digitizer Tablet	Statistical Analysis (ANOVA)	Relies on aggregated global statistics; ignores fine-grained temporal dynamics.
[16]	Drotár et al. (2016)	Digitizer Tablet	SVM on Kinematic Features	Dependent on manual feature engineering; lower accuracy on subtle symptoms.
[17]	Pereira et al. (2018)	Handwriting Image	CNN on Static Images	Discards dynamic information (velocity, pressure) by treating handwriting as images.
[11]	Cilia et al. (2022)	Tablet (DARWIN)	Benchmarking (Standard CNNs)	Standard CNNs struggle to capture long-term sequential dependencies.
[27]	Ahmed et al. (2025)	Synthetic (VAE)	Generative AI + XGBoost	Black box generation lacks physiological transparency; validation on small fixed split.

**Table 2:** List of Extracted Kinematic Channels.

Feature Group	Symbol	Channels	Description & Clinical Relevance
Raw Inertial	$a_{x},\;a_{y},\;a_{z}$	3	Linear acceleration (tremor intensity)
	$\omega_{x},\omega_{y},\omega_{z}$	3	Angular velocity from Gyroscope
Derived Linear	$v_{x},v_{y},v_{z}$	3	Reconstructed velocity (bradykinesia detection)
	$j_{x},j_{y},j_{z}$	3	Jerk (smoothness of movement)
Derived Angular	$\alpha_{x},\alpha_{y},\alpha_{z}$	3	Rotational acceleration (complex tremors)
Spatial	ϕ, θ, ψ	3	Roll, Pitch, Yaw (Pen orientation/posture)
Total		18	

**Table 3:** Hyperparameter search space and selected optimal values for baseline models.

Model	Hyperparameter	Search Space	Selected Optimal Value
Logistic Regression (LR)	Regularization (*C*)	{0.01, 0.1, 1, 10}	1.0
	Solver	liblinear	liblinear
Support Vector Machine (SVM)	Kernel	RBF	RBF
	Regularization (*C*)	{0.1, 1, 10}	10
	Gamma $\left(\gamma \right)$	{‘scale’, ‘auto’}	‘scale’
Random Forest (RF)	n_estimators	{50, 100}	100
	Max Depth	{5, 10}	10
k-Nearest Neighbors (k-NN)	Neighbors (k)	{3, 5, 7, 9}	5

**Table 4:** Layer by layer specification of the Hybrid CNN-BiLSTM architecture.

Layer Type	Output Shape	Parameters/Configuration
Input Layer	(1084, 18)	18 Kinematic Channels
Conv1D (Layer 1)	(1084, 64)	Filters: 64, Kernel: 7, Stride: 1, Pad: Same, Activation: ReLU
MaxPooling1D	(542, 64)	Pool Size: 2
Batch Normalization	(542, 64)	Momentum: 0.99, Epsilon: 0.001
Conv1D (Layer 2)	(542, 128)	Filters: 128, Kernel: 5, Stride: 1, Pad: Same, Activation: ReLU
MaxPooling1D	(271, 128)	Pool Size: 2
Batch Normalization	(271, 128)	Momentum: 0.99, Epsilon: 0.001
Bidirectional LSTM	(256)	Units: 128, Return Sequences: False, Merge: Concat
Batch Normalization	(256)	Momentum: 0.99, Epsilon: 0.001
Dense (Fully Connected)	(128)	Neurons: 128, Activation: ReLU
Dropout	(128)	Rate: 0.5
Output Layer	(1)	Neurons: 1, Activation: Sigmoid

**Table 5:** Summary of Results.

Model	Accuracy	Precision	Recall	F1-Score	AUC
Logistic Regression (LR)	0.71	0.68	0.65	0.66	0.747
Support Vector Machine	0.73	0.70	0.68	0.69	0.76
Random Forest (RF)	0.72	0.71	0.66	0.68	0.748
k-Nearest Neighbors (kNN)	0.59	0.56	0.53	0.54	0.617
CNN + Bidirectional LSTM	0.91	0.89	0.93	0.91	0.96

## Data Availability

The data could be requested from authors directly via email.

## Abbreviations

The following abbreviations are used in this manuscript:

**Acronyms**	**Full form**
AD	Alzheimer’s disease
ADNI	Alzheimer’s disease neuroimaging initiative
AI	Artificial Intelligence
AUC	Area under the ROC curve
BiLSTM	Bidirectional Long-Short Term Memory
CN	Cognitive Normal
CNN	Convolutional neural network
DARWIN	Diagnosis AlzheimeR WIth haNdwriting
EEG	Electroencephalography
kNN	k-nearest neighbors
LR	Logistic Regression
LSTM	Long-short term memory
MCI	Mild cognitive impairment
ML	Machine Learning
MRI	Magnetic resonance imaging
NC	Normal Control
PET	Positron emission tomography
RF	Random forest
ROC	Receiver operating characteristic
ROI	Region of interest
SVM	Support vector machines

## References

[1] World Health Organization (2023). Dementia Fact Sheet.

[2] Bell S.M., Barnes K., De Marco M., Shaw P.J., Ferraiuolo L., Blackburn D.J., Venneri A., Mortiboys H. (2021). Mitochondrial Dysfunction in Alzheimer’s Disease: A Biomarker of the Future?. Biomedicines.

[3] (2024). Alzheimer’s Association Report. 2024 Alzheimer’s disease facts and figures. Alzheimer’s Dement. J. Alzheimer’s Assoc..

[4] Khan I.J., Amin M.F.B., Deepu M.D.S., Hira H.K., Mahmud A., Chowdhury A.M., Islam S., Mukta M.S.H., Shatabda S. (2025). Alzheimer’s Disease Neuroimaging Initiative. Enhanced ROI guided deep learning model for Alzheimer’s detection using 3D MRI images. Inform. Med. Unlocked.

[5] Rallabandi V.P.S., Tulpule K., Gattu M. (2020). Alzheimer’s Disease Neuroimaging Initiative. Automatic classification of cognitively normal, mild cognitive impairment and Alzheimer’s disease using structural MRI analysis. Inform. Med. Unlocked.

[6] Bente L.-M., Himstedt L., Kacprowski T. (2025). Alzheimer’s Disease Neuroimaging Initiative. Domain specific transfer learning and classifier chains in Alzheimer’s disease detection using 3D convolutional neural networks. Inform. Med. Unlocked.

[7] Li Z., Wang H., Song J., Gong J. (2025). Exploring Task-Related EEG for Cross-Subject Early Alzheimer’s Disease Susceptibility Prediction in Middle-Aged Adults Using Multitaper Spectral Analysis. Sensors.

[8] Santos Toural J.E., Montoya Pedrón A., Marañón Reyes E.J. (2021). A new method for classification of subjects with major cognitive disorder, Alzheimer type, based on electroencephalographic biomarkers. Inform. Med. Unlocked.

[9] Sugden R.J., Diamandis P. (2023). Generalizable electroencephalographic classification of Parkinson’s disease using deep learning. Inform. Med. Unlocked.

[10] Aditya C.R., Pande M.B.S. (2017). Devising an interpretable calibrated scale to quantitatively assess the dementia stage of subjects with Alzheimer’s disease: A machine learning approach. Inform. Med. Unlocked.

[11] Cilia N.D., De Gregorio G., De Stefano C., Fontanella F., Marcelli A., Parziale A. (2022). Diagnosing Alzheimer’s disease from on-line handwriting: A novel dataset and performance benchmarking. Eng. Appl. Artif. Intell..

[12] Sourkatti H., Asuroglu T., Itkonen M., Alahäivälä A.-L.I., Tolppanen A.-M., Ihalainen J., Forsberg M.M. (2025). A comparative feature selection study: Predicting Alzheimer’s disease using primary healthcare and social services data. Inform. Med. Unlocked.

[13] Assaduzzaman M., Dutta M., Saha A., Paul S.G. (2024). ALSA-3: Customized CNN model through ablation study for Alzheimer’s disease classification. Inform. Med. Unlocked.

[14] Werner P., Rosenblum S., Bar-On G., Heinik J., Korczyn A.D. (2006). Handwriting process variables discriminating mild Alzheimer’s disease and mild cognitive impairment. J. Gerontol. Ser. B.

[15] Rosenblum S., Samuel M., Zlotnik S., Erikh I., Schlesinger I. (2013). Handwriting as an objective tool for Parkinson’s disease diagnosis. J. Neurol..

[16] Drotár P., Mekyska J., Rektorová I., Masarová L., Smékal Z., Faundez-Zanuy M. (2016). Evaluation of handwriting kinematics and pressure for differential diagnosis of Parkinson’s disease. Artif. Intell. Med..

[17] Pereira C.R., Pereira D.R., Rosa G.H., Albuquerque V.H.C., Weber S.A.T., Hook C., Papa J.P. (2018). Handwritten dynamics assessment through convolutional neural networks: An application to Parkinson’s disease identification. Artif. Intell. Med..

[18] Kourtis L.C., Regele O.B., Wright J.M., Jones G.B. (2019). Digital biomarkers for Alzheimer’s disease: The mobile/wearable devices opportunity. NPJ Digit. Med..

[19] Bazarbekov I., Razaque A., Ipalakova M., Yoo J., Assipova Z., Almisreb A. (2024). A review of artificial intelligence methods for Alzheimer’s disease diagnosis: Insights from neuroimaging to sensor data analysis. Biomed. Signal Process. Control.

[20] Park H., Shin S., Youm C., Cheon S.M. (2024). Deep learning-based detection of affected body parts in Parkinson’s disease and freezing of gait using time-series imaging. Sci. Rep..

[21] Babu K.K., Lukka S., Shabarish P., Sai A.L., Goud B.S.V., Yeshwanth G. (2025). Online signature verification using deep learning. Proceedings of the 2025 4th International Conference on Sentiment Analysis and Deep Learning (ICSADL).

[22] Kang L., Zhang X., Guan J., Huang K. (2024). Early Alzheimer’s disease diagnosis via handwriting with self-attention mechanisms. J. Alzheimer’s Dis..

[23] Ordóñez F.J., Roggen D. (2016). Deep Convolutional and LSTM Recurrent Neural Networks for Multimodal Wearable Activity Recognition. Sensors.

[24] El Maachi I., Bilodeau G.-A., Bouachir W. (2020). Deep 1D-Convnet for accurate Parkinson disease detection and severity prediction from gait. Expert Syst. Appl..

[25] Qi H., Zhu X., Ren Y., Zhang X., Tang Q., Zhang C., Lang Q., Wang L. (2024). A study of assisted screening for Alzheimer’s disease based on handwriting and gait analysis. J. Alzheimer’s Dis..

[26] Yegemberdiyev T., Daineko Y., Bazarbekov I. (2025). Efficiency of artificial intelligence in the diagnosis of cognitive disorders. Procedia Comput. Sci..

[27] Ahmed N., Hao Y., Yu C., Jin Z. (2025). Enhancing Alzheimer’s detection: VAE-augmented handwriting analysis. CCF Trans. Pervasive Comput. Interact..

[28] Alberdi A., Aztiria A., Basarab A. (2016). On the early diagnosis of Alzheimer’s Disease from multimodal signals: A survey. Artif. Intell. Med..

[29] World Medical Association (2013). World Medical Association Declaration of Helsinki: Ethical principles for medical research involving human subjects. JAMA.

[30] InvenSense (2016). MPU-9250 Product Specification: Revision 1.1.

[31] Liechti C. (2020). pySerial (Version 3.5) [Python Library]. https://github.com/pyserial/pyserial/.

[32] Mahony R., Hamel T., Pflimlin J.M. (2008). Nonlinear complementary filters on the special orthogonal group. IEEE Trans. Autom. Control..

[33] Hekmatmanesh A., Wu H., Jamaloo F., Li M. (2020). A combination of CSP-based method with soft margin SVM classifier and generalized RBF kernel for imagery-based brain–computer interface applications. Multimed. Tools Appl..

[34] Qi X., Li Q., He H., Guo Y. (2020). Random forest for bioinformatics. WIREs Data Min. Knowl. Discov..

[35] Lew C.O., Zhou L., Mazurowski M.A., Doraiswamy P.M., Petrella J.R. (2023). Alzheimer’s Disease Neuroimaging Initiative. MRI-based Deep Learning Assessment of Amyloid, Tau, and Neurodegeneration Biomarker Status across the Alzheimer Disease Spectrum. Radiology.

[36] Bazarbekov I., Ipalakova M., Daineko Y., Mukhanov S., Bazarbekova M., Zholdassova Z., Turgunova A., Kapyshev G. (2025). Design of a smart handwriting tool for early detection of Alzheimer’s disease. Proceedings of the 2025 International Conference on Artificial Intelligence, Computer, Data Sciences and Applications (ACDSA).

